# Novel Insights into Non-Invasive Diagnostic Techniques for Cardiac Amyloidosis: A Critical Review

**DOI:** 10.3390/diagnostics14192249

**Published:** 2024-10-09

**Authors:** Marco Maria Dicorato, Paolo Basile, Giuseppe Muscogiuri, Maria Cristina Carella, Maria Ludovica Naccarati, Ilaria Dentamaro, Marco Guglielmo, Andrea Baggiano, Saima Mushtaq, Laura Fusini, Gianluca Pontone, Cinzia Forleo, Marco Matteo Ciccone, Andrea Igoren Guaricci

**Affiliations:** 1 Interdisciplinary Department of Medicine, University of Bari “Aldo Moro”, Polyclinic University Hospital, 70124 Bari, Italy; mm.dicorato@gmail.com (M.M.D.); paolo.basile@uniba.it (P.B.); m.c.carella92@gmail.com (M.C.C.); marialudovica97@libero.it (M.L.N.); ilaria.dentamaro@gmail.com (I.D.); cinzia.forleo@uniba.it (C.F.); marcomatteo.ciccone@uniba.it (M.M.C.); andreaigoren.guaricci@uniba.it (A.I.G.); 2 Department of Radiology, IRCCS Istituto Auxologico Italiano, San Luca Hospital, 20149 Milan, Italy; 3 Department of Cardiology, Division of Heart and Lungs, Utrecht University, Utrecht University Medical Center, 3584 Utrecht, The Netherlands; m.guglielmo@umcutrecht.nl; 4 Department of Cardiology, Haga Teaching Hospital, 2545 The Hague, The Netherlands; 5 Department of Perioperative Cardiology and Cardiovascular Imaging, Centro Cardiologico Monzino, IRCCS, 20138 Milan, Italy; andrea.baggiano@cardiologicomonzino.it (A.B.); saima.mushtaq@cardiologicomonzino.it (S.M.); laura.fusini@cardiologicomonzino.it (L.F.); gianluca.pontone@cardiologicomonzino.it (G.P.); 6 Department of Biomedical, Surgical and Dental Sciences, University of Milan, 20122 Milan, Italy

**Keywords:** cardiac amyloidosis, cardiomyopathies, magnetic resonance imaging, echocardiography, radionuclide imaging, cardiac imaging techniques, artificial intelligence

## Abstract

Cardiac amyloidosis (CA) is a cardiac storage disease caused by the progressive extracellular deposition of misfolded proteins in the myocardium. Despite the increasing interest in this pathology, it remains an underdiagnosed condition. Non-invasive diagnostic techniques play a central role in the suspicion and detection of CA, also thanks to the continuous scientific and technological advances in these tools. The 12-lead electrocardiography is an inexpensive and reproducible test with a diagnostic accuracy that, in some cases, exceeds that of imaging techniques, as recent studies have shown. Echocardiography is the first-line imaging modality, although none of its parameters are pathognomonic. According to the 2023 ESC Guidelines, a left ventricular wall thickness ≥ 12 mm is mandatory for the suspicion of CA, making this technique crucial. Cardiac magnetic resonance provides high-resolution images associated with tissue characterization. The use of contrast and non-contrast sequences enhances the diagnostic power of this imaging modality. Nuclear imaging techniques, including bone scintigraphy and positron emission tomography, allow the detection of amyloid deposition in the heart, and their role is also central in assessing the prognosis and response to therapy. The role of computed tomography was recently evaluated by several studies, above in population affected by aortic stenosis undergoing transcatheter aortic valve replacement, with promising results. Finally, machine learning and artificial intelligence-derived algorithms are gaining ground in this scenario and provide the basis for future research. Understanding the new insights into non-invasive diagnostic techniques is critical to better diagnose and manage patients with CA and improve their survival.

## 1. Introduction

Cardiac amyloidosis (CA) is the most common cardiac storage disease and is caused by the progressive extracellular deposition of misfolded proteins in the myocardium [1]. This disease leads to restrictive cardiomyopathy (CMP), heart failure (HF) (mainly with preserved ejection fraction), and arrhythmias. Among the nine amyloidogenic proteins demonstrated to accumulate in the heart, the two responsible for over 98% of cases are monoclonal immunoglobulin light chains (AL) and transthyretin (ATTR), either in its hereditary (ATTRv) or acquired (ATTRwt) form [2]. The introduction of increasingly advanced diagnostic tools, coupled with the growing interest in this pathology in recent years, has improved its recognition, although it still remains an underrecognized condition [3,4]. According to recent Guidelines for the management of CMPs, the diagnostic algorithm for CA begins with a suspicion in patients exhibiting an increased left ventricle wall thickness (LVWT) (≥12 mm), along with at least one red flag, either extracardiac or cardiac, including specific electrocardiographic, echocardiographic, and cardiac magnetic resonance (CMR) features [2]. Following this, hematologic tests (serum free light chain and serum and urine immunofixation) are conducted, along with planar and single-photon emission computed tomography (SPECT) with bone-seeking tracers. Subsequently, CMR (if not previously performed) and/or histological confirmation, if required, are indicated. A non-invasive diagnosis can only be made for ATTR-CA, for which genetic tests are also recommended, while histological confirmation is always necessary in AL-CA. ATTR-CA can be confirmed through typical echocardiographic and/or CMR findings in addition to SPECT grade 2 or 3 myocardial radiotracer uptake, after excluding a clonal dyscrasia [1,2]. Thus, it is crucial to understand the specific characteristics of this pathology, as well as new evidence and advances in non-invasive exams, in order to raise the right suspicion and correctly recognize this often-misdiagnosed condition, promptly initiating specific treatment and improving patient outcomes. The aim of this review is to shed light on the most recent progress and evolution in non-invasive techniques used for the diagnosis of CA.

## 2. Electrocardiography

The 12-lead electrocardiography (ECG) is a low-cost, simple, and reproducible exam, and although it is not included in non-invasive diagnostic criteria, it is an essential first-approach instrument that can raise the suspicion of CA [2].

One of the most reported ECG findings in CA patients is the reduction in QRS complex voltage, primarily in peripheral leads. Although this reduction is a well-known pattern present in up to 60% of affected people, several studies have shown its low accuracy also due to different diagnostic criteria used to assess it [5], as shown in Table 1. Low QRS voltages (LQRSV) are usually defined as voltages ≤ 0.5 mV in each limb lead and/or ≤1 mV in each pre-cordial lead. Additionally, the criterion of the Sokolow/Lyon index (the sum of S wave in V1 and R wave in V5 or V6) < 1.5 mV is commonly used [6]. The prevalence of this feature depends on the CA subtype and is higher in AL-CA according to numerous studies [5,6,7]. The reason for this difference is probably due to severe myocardial inflammation due to the cytotoxicity of light chains fibrils and/or peripheral tissue amyloid storage, which can cause higher electric impedance in the AL-CA [5]. This ECG feature was recently included in the T-Amylo prediction model along with widely available parameters (age, gender, carpal tunnel syndrome, and interventricular septum thickness) [8],. The obtained score was validated, showing a good diagnostic accuracy in predicting the likelihood of ATTR-CA, excluding the pathology in 30% of patients with left ventricular hypertrophy (LVH) without performing further tests [8]. LQRSV are also associated with advanced disease and a worse prognosis. In a multicenter study including 411 CA patients, LQRSV were an independent predictor for cardiovascular mortality, consistent with previous findings [5,7]. This evidence could be considered a consequence of more extensive fibril deposition and, in turn, a more extensive death of myocardial cells, resulting in a reduction in the electrical activity of the heart. LQRSV may even be due to pericardial effusion, a well-known sign of advanced disease, that can cause a reduction in QRS voltages even in the absence of CA, further limiting the accuracy of this ECG sign [5]. Thus, ESC Guidelines [2] suggest as a red flag for CA the disproportion between the electrocardiographic mass assessed by QRS voltages and LV thickness or LV mass measured by cardiac imaging. This parameter shows a higher sensitivity compared to LQRSV [6,9]. Similarly to LQRSV, a mass-to-voltage ratio is correlated with the progression of the pathology and hospitalizations for HF, as it reflects the burden of amyloid deposits [10].

In addition to the well-known signs described above, other ECG criteria and scoring systems were proposed in recent years [11,12,13]. In particular, there is the pseudo-infarction pattern, present in up to 70% of CA patients, which is defined as pathological Q waves or QS complexes in two consecutive leads, but without a previous occurrence of myocardial infarction or akinetic areas at imaging [7,14]. This feature results from the impairment of myocardial tissue and is consequently associated with a worse prognosis [15]. Other than this feature, it is not uncommon to observe fragmented QRS complexes (fQRS) [7], which are defined as “non-specific” abnormalities, including notches and various RsR′ patterns in the absence of QRS prolongation, presumed to be a consequence of myocardial scars [16]. The prevalence of fQRS is higher in patients with AL-CA, and there is evidence that this sign is independently associated with a poorer prognosis [17]. However, fQRS, as a result of an impaired myocardium, can also be found in other cardiovascular conditions, such as coronary artery disease, arrhythmogenic right ventricular CMP, and Brugada syndrome, limiting the specificity of this pattern [17]. Another recent study proposed five novel ECG criteria: QRS amplitude in lead I < 0.55 mV, QRS amplitude in lead aVR < 0.5 mV, average QRS amplitude of leads I + aVR < 0.575 mV, average QRS amplitude of leads I + aVR/average QRS amplitude of leads V1–4 < 0.375, and average QRS amplitude of leads I + aVR/longest time to the onset of intrinsicoid deflection in leads I, aVL, V1–6 < 0.0115 [11]. The former three parameters tested were also based on previous publications by Huang et al. [18]. These criteria not only showed better diagnostic accuracy than classical ECG criteria, with the exception of LQRSV in limb leads but it was also striking to see that they were comparable to the recommended and more acknowledged echocardiographic myocardial deformation criteria in diagnosing CA. ST-T segment changes are also frequent: in particular, T wave inversion and/or ST-segment depression are detected primarily in AL-CA, mainly in inferior and lateral leads, and must be differentiated from other causes of hypertrophic phenotype.

Electrocardiography is very useful in detecting supraventricular and ventricular arrhythmias and conduction disturbances, mainly caused by amyloid infiltration [19]. Sinus node dysfunction and conduction abnormalities, including atrioventricular blocks and intraventricular conduction delays, were reported in CA [20] and represent a frequent cause for pacemaker implantation in this population [21]. In particular, in ATTR-CA, left bundle branch block (BBB) is more frequent than other conduction impairments. Supraventricular arrhythmias are frequent, especially in ATTR-CA [22], and atrial fibrillation (AF) is more common than other types, such as atrial flutter, atrial tachycardia, and atrioventricular nodal reentry tachycardias, although these can also be found [23].

As the percentage of misdiagnosis and delayed diagnosis remains significant, machine learning algorithms were implemented in recent years, with deep-learning ECG models capable of detecting early pathological changes. Schrutka et al. [24] elaborated a machine learning diagnostic algorithm for the detection of CA based on surface ECGs through ECG-imaging techniques. In this algorithm, LQRSV were more predictive of CA if considered in inferior leads and with a cutoff value of 1 mV instead of 0.5 mV. Other features considered in this model were right BBB, left anterior fascicular block, and the delay of R-wave progression in leads V1-V3 [24]. More recently, Haimovich et al. [25] developed deep learning models based on 12-lead and single-lead ECGs to detect LVH and the cardiological diseases that cause it, including CA. These algorithms showed high performance even for the prediction of clinical outcomes. Moreover, the single-lead model also showed good accuracy, supporting its adoption in mobile devices, which are accessible to everyone [25].

The 12-lead ECG remains a key tool for the diagnosis of CA and should always be performed before any other imaging technique, also because of its low cost and time of execution. Furthermore, new evidence supports the use of novel ECG markers that were demonstrated to be very accurate in promptly identifying and following up the disease [26]. Due to the not high specificity of these ECG features, the application of artificial intelligence (AI) in the setting of CA could be very useful in recognizing the disease, even in the absence of advanced imaging tools [25].

## 3. Echocardiography

**Figure 1:**
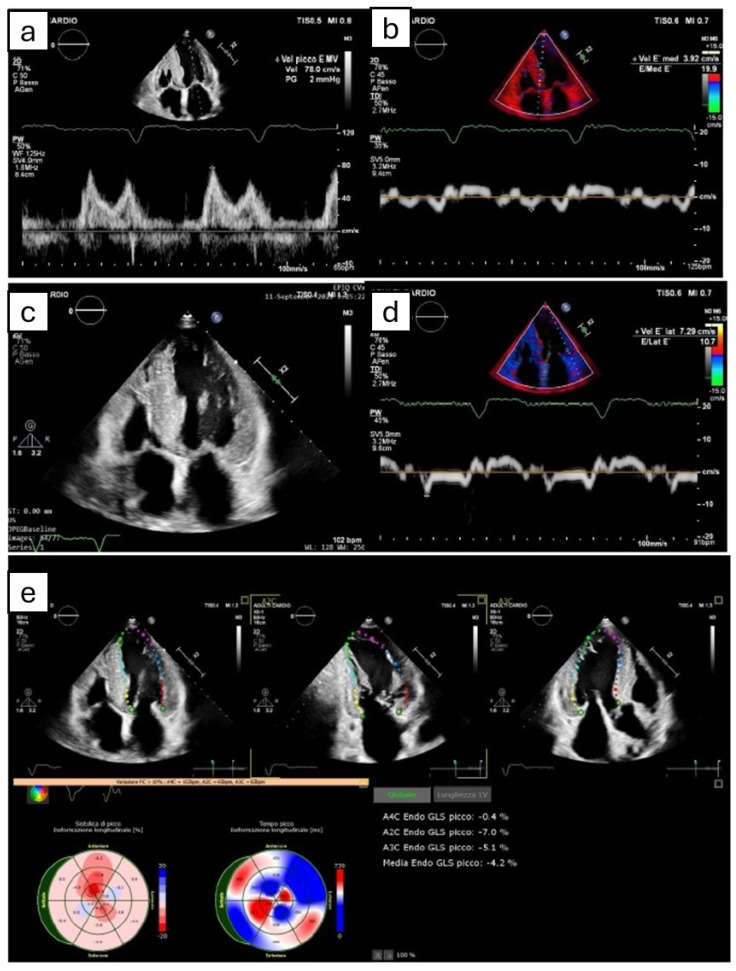
The figure shows the most common echocardiographic signs that can potentially suggest amyloidotic cardiomyopathy, even though none of them are pathognomonic. (**a**,**b**,**d**) Grade II diastolic dysfunction, with pseudonormal pattern at transmitral power Doppler (**a**) and reduced septal (**b**) and lateral (**d**) e’ velocity at tissue Doppler imaging. (**c**) Symmetric biventricular hypertrophy, with thickening of interatrial septum. (**e**) Apical sparing pattern derived by Speckle tracking echocardiography.

Echocardiography, with an assessment of both traditional (non-deformation) and advanced (strain-derived) parameters, is essential in raising suspicion for CA and is usually the first-line imaging technique (Figure 1) [27]. According to recent ESC Guidelines [2], LVWT ≥ 12 mm is a mandatory parameter in order to start the screening path for CA, whereas reduced longitudinal strains with apical sparing and decreased QRS voltage compared to mass ratio are two red flags included in the subsequent algorithm [2]. Several echocardiographic features were described in these patients, although none of them are specific for CA. In particular, we mentioned the thickening of ventricular walls, interatrial septum, and valves due to the amyloid deposition, along with biatrial enlargement and restrictive LV filling [27]. Pericardial effusion and valvular insufficiency can also be present and are associated with a more severe prognosis [28]. Specifically, increased thicknesses are more evident in ATTRwt than ATTRv or AL, likely due to the longer duration of the pathology [27].

### 3.1. Left Ventricle

Amyloid storage results in restrictive CMP and consequent symptoms of HF with preserved left ventricular ejection fraction (LVEF). It is acknowledged that CA causes systolic dysfunction only in the late phase of the disease, whereas LVEF is often ≥50% and sometimes also increased (≥65%) [29]. A recent study reported a CA prevalence of 28.6% among patients with hypertrophic, non-dilated hearts with preserved LVEF and at least one echocardiographic red flag of pathology, achieving an accuracy above 70% with two or more red flags [30,31]. An LV deformation assessed through a global longitudinal strain (GLS) at two-dimensional speckle tracking echocardiography (STE) is assuming an increasingly important role in evaluating LV function, as it can detect functional myocardial dysfunction, which usually occurs before LVEF impairment due to increased myocardial stiffness. The apical sparing pattern, described by Phelan et al. in 2012, is a well acknowledged feature, even though its mechanism is poorly understood [27,32]. It is commonly defined as a “bull’s eye” or “cherry on top” plot [33]. We underline that, while not pathognomonic, this feature can suggest the presence of CA, differentiating it from other hypertrophic phenotypes [34]. Wali et al. recently evaluated the predictive value of apical sparing at STE in both AL and ATTR-CA patients, concluding that it was present in only one-third of patients and was more accurate in older patients with hypertrophic LV walls [33]. Thus, apical sparing is not diagnostic but provides an indication for the execution of further tests. Even in this case, an impaired GLS was demonstrated to be an independent predictor of mortality in CA patients [27,35]. Recent studies have demonstrated how an LV strain measured in AL-CA patients has a greater prognostic significance than other echocardiographic findings and can predict the worsening of cardiac involvement. Useful strain data can be obtained from all four cardiac chambers, and each of them have prognostic associations with survival [36]. Myocardial work (MW) indices are other useful parameters that have emerged in recent years. Unlike GLS, they consider myocardial systolic deformation in relation to afterload by estimating intraventricular pressure during a cardiac cycle [37]. Actually, afterload is altered in CA due to the restrictive evolution, and this alteration may lead to a misinterpretation of GLS measures [38]. Roger-Rollè et al. demonstrated in 118 CA patients that MW indices were well correlated with usual prognosis markers such as N-terminal pro-brain natriuretic peptide (NT-proBNP), glomerular filtration rate (eGFR), and troponin and were more accurate than LVEF and MCF, but not than GLS, in predicting mortality [38]. A recent study enrolling 61 AL-CA patients also demonstrated the role of MW indices in predicting response to hematological therapy at a 1-year follow-up. A significant reduction in NT-proBNP and posterior wall thickness, associated with an increase in GWI and GLS, was reported, more pronounced in patients with a complete response to treatment [39]. Moreover, patients with GLS and GWI improvement had a better prognosis, and these two parameters resulted as independent predictors of survival [39]. Importantly, the prognostic value of each echocardiographic marker of LV function may vary according to the stage of the disease [40].

Other echocardiographic techniques sought to demonstrate a significant diagnostic and prognostic role in CA. Myocardial contraction fraction (MCF) is a three-dimensional echocardiographic volumetric measure of myocardial shortening, given by the ratio of stroke volume to myocardial volume. It does not depend on chamber geometry, and several studies demonstrated its accuracy in distinguishing physiological from pathological hypertrophy, as in the former, shortening is increased [41]. MCF can even be calculated through two-dimensional guided M-mode echocardiography. Maurer et al. demonstrated in a population with a normal EF that it is independently associated with negative cardiovascular outcomes [42]. Furthermore, MCF was found to predict mortality better than LVEF in both AL-CA and ATTR [43]. The Stroke Volume (SV) index is a widely available parameter and has been demonstrated to predict outcomes in AL-CA patients with a comparable accuracy, and in one study even superior accuracy to MCF and longitudinal strain [44]. Slostad et al. first used novel pixel intensity quantification software ImageJ, publicly available and developed by the National Institute of Health (http://imagej.nih.gov./ij/docs/guide), to differentiate ATTR-CA from AL-CA [45]. In fact, ATTR fibrils containing microcalcifications are preferentially located in the interventricular septum. The authors specifically studied the septal reflectivity ratio, calculated as the mean pixel intensity of the visible anterior septal wall divided by the mean pixel intensity of the visible posterior lateral wall in the end-diastolic parasternal long-axis. A value > 1.23 was indicative of ATTR-CA over AL-CA, with even better accuracy than other strain-derived measures in differentiating the two main subtypes of CA.

### 3.2. Left Atrium

The left atrium (LA), despite often being overlooked, is an important component of the heart implicated in cardiac performance, thromboembolic risk, and the genesis of arrhythmias [46]. Although studies about CA have focused mainly on the consequences of amyloid storage throughout the LV [47], the involvement of LA alone or in addition to the right atrium is common in this disease [48]. Traditionally, LA function has been related to its anatomical enlargement, which has been demonstrated to be a poor prognostic marker [49] not only in CA patients but also in many other conditions [50]. Similarly to LV, changes in LA function, which can be assessed by STE, usually precede anatomical changes. Nochioka et al. demonstrated that an LA strain in the three phases (reservoir, conduit, and contraction) is compromised for both AL-CA and ATTR-CA (especially for ATTRwt), and the greater the impairment, the greater the decrease in LV systolic and diastolic performance, independently from LA size, suggesting a parallel infiltration of the LA and LV by amyloid [47]. According to the authors, the more severe depletion of LA function in ATTRwt-CA compared to AL-CA was due to the slower progression of amyloid deposition [47]. Bandera et al. [51] studied 906 ATTR-CA patients through LA-STE, demonstrating that increased atrial stiffness, with an impairment of the three phases of atrial function, was independently associated with prognosis, as well as the atrial fibrillation and atrial electromechanical dissociation with the absence of atrial contraction [51]. These recent findings support a more extensive use of LA strain not only for earlier recognition of the pathology but also for better prognostic stratification.

### 3.3. Right Heart

The role of the right heart is central in all cardiovascular diseases, including CA [52]. Several studies demonstrated the frequent involvement of the right ventricle (RV) in both AL-CA and ATTR-CA and how its dysfunction is related to worse prognosis [53,54,55,56,57]. It is acknowledged that CA patients exhibit altered echocardiographic RV parameters, such as increased RV size, basal diameter, and wall thickness, as well as a dilated inferior vena cava and right atrium (RA), leading to a reduced systolic function, usually assessed by a tricuspid annular plane systolic excursion (TAPSE) and TDI [55]. Parameters such as a reduced septal E′ and tissue velocity peak of the RV are also indicative of dysfunction [58]. Additionally, a reduced longitudinal RV free wall strain has been described in CA, with an independent association with poor outcomes [56,59,60,61]. Tricuspid regurgitation (TR) is often present due to both amyloid valvular deposition and RV remodeling and dilation caused by increased LV filling pressure [53]. There is evidence that even the RA is impaired in CA, with progressive dilation and the loss of contractile properties [62]. Because of these recent findings, we suggest systematically performing, if possible, a complete assessment of right heart echocardiographic parameters in patients affected by or suspected of CA.

## 4. Cardiac Magnetic Resonance

CMR has acquired an increasingly central role in the diagnostic pathway of many pathologies, particularly in the setting of cardiomyopathies [2,63,64,65,66,67]. CMR is considered the gold standard for the morphological characterization of the four cardiac chambers and provides high-definition images associated with tissue characterization, representing an in vivo biopsy [27].

### 4.1. Late Gadolinium Enhancement

Myocardial LGE usually indicates fibrosis areas (e.g., due to inflammation, ischemic processes, and more), whereas in the case of CA, LGE corresponds to interstitial deposits of amyloid [68,69,70]. For this reason, LGE is present in almost all cases of CA, more frequently in the ATTR subtype, and the more its extension, the worse the prognosis [71]. A recent meta-analysis of 257 patients concluded that LGE had a sensitivity of 85% and a specificity of 92% for CA diagnosis [72]. The characteristic LGE pattern is global and mainly subendocardial, above all for AL-CA, but transmural or patchy LGE can also be found [73]. The failure to null the myocardium on the inversion scout sequence performed before the gadolinium administration is considered a pathognomonic sign. Since LGE is not easy to quantify in CA due to the wide range of patterns and signal intensities, it is poorly related to progression of the disease and cannot be considered a reliable tool in order to assess amyloid changes over time [27]. This limit is overcome by T1 mapping, a newer technique able to identify amyloid deposits earlier than LGE.

### 4.2. T1 Mapping and Extracellular Volume Fraction

**Figure 2:**
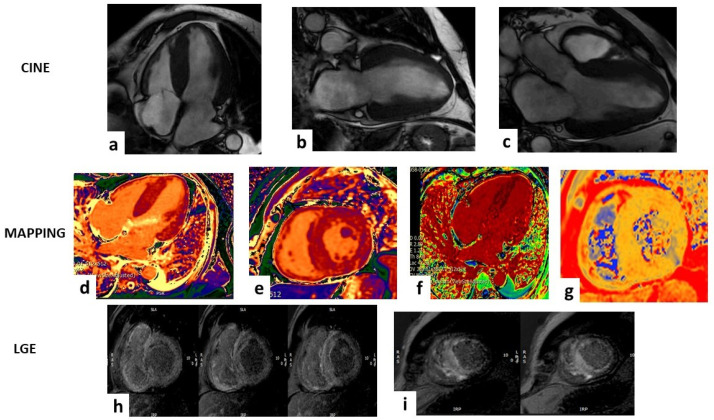
Case of a 63-year-old male that showed a hypertrophic left ventricle (LV) in the steady-state free-precession sequences (SSFP) in (**a**–**c**). The mapping sequences showed high values of T1 mapping (**d**,**e**), very high extracellular volume (**f**), and mild high value of T2 mapping (**g**). Late gadolinium enhancement sequences (LGE) (**h**,**i**) showed abnormal gadolinium kinetics with a faster washout of gadolinium from myocardium and blood pool and a transmural pattern in both ventricles. All these findings are suggestive for ATTR amyloidosis.

CMR T1 values are significantly elevated in CA, more so than in all other cardiomyopathies, making them very useful in differential diagnosis [74,75,76]. Native T1 mapping, which is measured before gadolinium administration, identifies abnormalities in myocardial cells and/or extracellular space. These abnormalities may correspond to areas of edema, fibrosis, or extracellular expansion seen in CA (Figure 2). Similar to LGE, native T1 values are elevated in regions with amyloid deposits and are even more sensitive than LGE in the early detection of CA. Ioannou et al. recently demonstrated that changes in native T1 values effectively track treatment response in AL-CA patients and are independently associated with mortality [77]. Native T1 values also reflect variations in ECV and T2 values, in addition to NT-proBNP levels. However, native T1 mapping suffers from low reproducibility due to its dependence on specific machines and magnetic fields [78]. On the other hand, post-contrast T1 mapping and an Extracellular Volume Fraction (ECV) evaluation, performed after gadolinium administration, are more standardized. These measurements rely solely on extracellular space and are not affected by variations in myocardial cells [27]. Similar to LGE and T1 values, ECV—which requires pre- and post-contrast T1 Mapping associated with hematocrit—is significantly higher in CA than in normal tissue and correlates with disease severity [27] even better than LGE and pre- and post-contrast T1 values alone [79,80]. Liu et al. demonstrated that native T1 and ECV are independent predictors of mortality in CA patients, in agreement with past studies [79,81,82]. A recent meta-analysis including 955 patients has shown that a higher ECV and T1 times and lower T2 to skeletal muscle ratio were associated with higher mortality [83]. Furthermore, ECV is a good marker in assessing response to therapy and overall disease burden, especially for AL-CA [84,85]. Among the others, Briasoulis et al. showed that non-LGE CMR traces a characteristic profile in CA, differentiating it from other causes of LVH and with a significant association with other acknowledged prognostic markers such as Mayo stage and NT-proBNP. This property reinforces evidence about the diagnostic and prognostic power of T1 mapping and ECV [86]. In the end, the possibility of using ECV for spleen assessment has emerged to differentiate between AL- and ATTR-CA—in which the involvement of this organ is rare—with encouraging results [86,87].

### 4.3. T2 Mapping

Transverse relaxation time (T2) has received less attention compared to T1 mapping and ECV in the setting of CA. This parameter reflects the status of myocardial water and thus the edema associated with amyloid toxicity, which is more characteristic of AL-CA [88]. Consequently, T2 times are increased in both AL- and ATTR-CA, but its prognostic significance and its correlation with disease activity and with patient survival have only been demonstrated for the AL subtype [86,88]. Based on these findings, a model capable of distinguishing AL-CA from ATTR-CA was recently proposed, incorporating three parameters: age, RV ejection fraction, and a mean T2 value [89]. Additionally, Briasoulis et al. found that T2 values in CA patients were higher not only compared to controls but also compared to patients affected by LVH and Aortic Stenosis (AS), indicating a significant association of this parameter with CA [86]. Given these promising results, a systematic use of T2 sequences during CMR examinations in CA patients should be encouraged.

### 4.4. Emerging Techniques

The diffusion tensor CMR (DT-CMR) is a novel non-contrast CMR technique that assesses alterations in myocardial microstructure by measuring water diffusion through tissues [90]. This method allows the quantification of parameters such as fractional anisotropy, mean diffusivity, and myocardial sheetlet orientation. Preliminary studies have demonstrated a good correlation of these parameters with ECV and T1 mapping. Despite the first promising results, more research is needed to validate and standardize this interesting tool [90]. CMR feature tracking (CMR-FT) is a technique used for the assessment of myocardial deformation, based on steady-state free-precession sequences [91,92]. Torsion is the twisting motion of LV around its long axis caused by the contraction of myocardial cells in the LV wall [93]. Through LV torsion mechanic analyses acquired by CMR cine images, it is possible to quantify the LV diastolic and systolic function with greater accuracy and reproducibility than STE [94]. Recently, Zheng et al. demonstrated a significant decrease in global torsion, base-mid torsion, and the peak diastolic torsion rate in AL-CA patients with preserved LVEFs, compared to controls, with increasing impairment as systolic dysfunction progresses [93]. Thus, similar to strain, LV torsion impairment spares the apex. Furthermore, in the same study, LV global torsion was related to the LGE pattern, with the potential—according to the authors—of distinguishing transmural LGE from non-transmural LGE. Lastly, RV end-systolic volume and RVEF were independently associated with torsion parameters, supporting the influence of RV function on torsion mechanisms [93]. Finally, we must mention radiomics as a newborn approach that consists of converting imaging features into quantitative parameters that are then analyzed by various computer programs, including AI tools, capable of collecting data that cannot be identified by human eyes [95,96,97]. Zhou et al. first studied LGE-based radiomic features in CA patients, elaborating the Rad-Score, which showed better prognostic performance than LGE quantitative and semi-quantitative parameters [98].

## 5. Nuclear Imaging Techniques

### 5.1. Bone Scintigraphy and SPECT/CT

Several studies have focused on nuclear imaging in CA, and its cornerstone role in this setting is widely recognized (Table 2) [99]. A large meta-analysis comparing CMR, positron emission tomography, and SPECT for CA diagnosis concluded that SPECT has the best diagnostic performance [100]. The 2023 ESC Guidelines consider bone scintigraphy or SPECT as mandatory exams in the diagnostic algorithm of CA [2]. In addition to hematologic tests, they can eventually lead to non-invasive diagnosis of ATTR-CA, even without performing a biopsy [2]. According to the authors, tomographic acquisition should be preferred to reduce the possibilities of misclassification [2]. Despite a poor comprehension of the mechanism of myocardial uptake of the drug, probably associated with calcium deposits, the Perugini score, a visual—from zero to three—grading system based on the quantification of myocardial uptake compared to nearby rib uptake, is decisive in the choice of the following diagnostic pathway [101]. Since up to one in five patients affected by AL-CA shows a positive bone scintigraphy as well [102], a heart to contralateral lung (H/CL) uptake ratio of >1.5 at 1 h from the tracer administration was studied, demonstrating a high accuracy in distinguishing the two main types of CA [103]. Furthermore, an H/CL ratio >1.5 was associated with worse prognosis in ATTR-CA patients [103]. Guo et al. found that a cutoff value of 1.51 for the H/CL ratio was more efficient than the visual score in the diagnosis of ATTRv-CA [104]. Recent studies agree on setting the optimal time point at 3 h after tracer administration to minimize blood pooling and false-positive results [105,106]. There is evidence that a low uptake of radiotracer can be present not only in AL-CA but also in some ATTR variants, such as Phe64Leu, Glu61Ala, or Val50Met [107,108], making SPECT not always a reliable tool if considered alone. Other semi-quantitative scores were studied, such as the heart-to-whole-body ratio (H/WB) and the heart-to-pelvis ratio (H/P) [109,110]. These are simple and more reproducible than the Perugini score but need further validation [110]. The main disadvantage of planar and conventional SPECT imaging is the blood pooling in the LV cavity, which masks the myocardial wall uptake and increases the number of false-positive scans. The association of SPECT with computed tomography (CT) can overcome this problem. Al Taha et al. recently reported their experience with fusion SPECT/CT imaging, highlighting a reduction in equivocal and false-positive cases when it was performed as a standard practice, with a great impact on the final diagnosis of CA [111]. In total, 73% of cases were reclassified, suggesting that SPECT should always be performed after the acquisition of planar imaging, and possibly with the fusion SPECT/CT technique, to achieve the best diagnostic accuracy [109,111]. Mallón Araujo et al. [112] validated the need for SPECT/CT to complete planar acquisition, demonstrating that through the quantitative estimation of amyloid burden (DPD load), a higher diagnostic accuracy is reached. These recent findings could be pivotal in reducing false positives, even due to the key role that bone scintigraphy has recently assumed in the non-invasive diagnosis of ATTR-CA, but further comparative research on a larger sample of patients is needed [113].

The Perugini score and semi-quantitative amyloid evaluation methods have some limitations: in addition to their non-quantitative nature, they are operator-dependent and are based on the comparison with other body sites, leading to a possible misinterpretation when the patient also shows abnormal extracardiac uptake. The development and standardization of nuclear imaging have led to a more extensive use of quantitative SPECT measurements, with a precise estimation of amyloid burden [122]. Recently, Papathanasiou et al. performed 99mTc-DPD scintigraphy before tafamidis initiation and after at least 9 months [123]. They demonstrated a regression of mean H/CL and SUV max, with a decrease in Perugini grade in five patients, whereas no changes in NT-proBNP or echocardiographic features were reported. This study showed the potential of SPECT-derived parameters in the assessment of disease activity and response to treatment [123]. Further proof of this potential was given by a larger study including 40 ATTR-CA patients, which demonstrated a significant reduction in the SUV retention index after treatment with tafamidis, parallel to improvements in NT-proBNP, LA volume index, LV global longitudinal strain, LVEF, LV cardiac index and RV functions [124]. On the other hand, Tingen et al. studied the outcomes of 20 ATTRv-CA patients undergoing therapy with patisiran and 12 with transthyretin stabilizers, finding a reduction in tracer myocardial uptake in the former group, in contrast to an uptake increase detected in the latter population, suggesting the progression of the pathology [115]. Compared to traditional follow-up parameters, bone scintigraphy resulted in more sensitivity in monitoring the disease evolution and treatment response, with the future perspective of its application for the differentiation between responder and non-responder patients [115,116]. Although some studies were published, there is still no agreement on a possible prognostic role of nuclear imaging data in CA. Vranian et al. found a significant correlation of quantitative and semiquantitative Tc99m-PYP scintigraphy parameters with all-cause mortality or HF hospitalization in subjects with suspected ATTR-CA but not in those with confirmed disease [125]. Martyn et al. demonstrated a low impact of tracer uptake degree on clinical outcomes, in contrast to a left ventricular uptake, which is supposed to be more associated with worse prognosis [126,127]. Similar to echocardiography and CMR, a decreased TcPYP uptake in the apical segments of LV was found by Sperry et al., further supporting an apical sparing pattern as a typical trait of CA. In this study, the regional distribution of an LV tracer uptake was related to mortality, which is different from H/CL ratio and total LV uptake [127]. Regarding the exact localization of the radiotracer in the heart, its accumulation mainly in LV is acknowledged [114,128]. Recently, a multicenter study [117] analyzed SPECT images of 1422 patients affected by ATTR-CA, finding an uptake of the radiotracer in RV, in addition to LV, in 100% of cases. In this large cohort, unlike the Perugini score, diffuse RV uptake was independently associated with all-cause mortality compared to focal RV uptake. The aforementioned research raises strong awareness about the use of SPECT tracer uptake quantification in RV as new prognostic marker in ATTR-CA [117].

### 5.2. Positron Emission Tomography

Since mainly transthyretin shows bone-avid tracer uptake, awareness has been raised about the use of positron emission tomography (PET) with drugs that directly target amyloid substance regardless of the specific protein, enabling an earlier detection of the pathology [129]. According to a recent meta-analysis, the sensitivity and specificity of this technique for the diagnosis of CA are 95% and 98%, respectively, with semiquantitative parameters performing particularly well for the AL subtype [130]. 18F-labeled radiotracers (18F-florbetapir, 18F-florbetaben, and 18F-flutemetamol) demonstrate myocardial uptake on both early and late scans for AL-CA, whereas it is primarily on early images for ATTR-CA [118]. The ultrastructural similarity of these tracers to thioflavin gives them the capacity to bind to the beta-pleated amyloid fibrils. 11C-Pittsburg compound B (PIB) was also studied, with encouraging results [119,131]. In the meantime, other radiotracers are under study. The 123I-labeled serum amyloid P component (123I-SAP) binds indiscriminately to all types of amyloid proteins, being able to evaluate the burden of systemic disease, but it hardly penetrates heart tissue, limiting its use in the cardiological setting [132]. 124I-evuzamitide is another novel tracer able to identify systemic amyloid deposits through its affinity with glycosaminoglycans, which are present in high quantities in amyloid fibrils [133]. 68Gallium-labeled fibroblast activation protein inhibitor (68Ga-FAPi) was demonstrated to permeate the thickened wall of the LV in AL-CA patients [134]. A fusion PET/MRI imaging tool was also described, with the possibility of better characterization of tissue features and morphologic measurements [129]. In the end, deep learning algorithms can be helpful in this context as well. Delbarre et al. developed a bone scintigraphy model for the detection of myocardial uptake with a Perugini grade ≥ 2 [98]. Similarly, Bhattaru et al. recently validated a highly accurate deep learning-based tool for ATTR-CA diagnosis using bone scintigraphy and SPECT/CT imaging [135]. A deep learning model based on 18F-florbetaben PET images has also demonstrated good performance in differentiating AL- from ATTR-CA [136].

### 5.3. Cardiac Innervation Imaging

Amyloid substance can deposit into conduction tissue, causing the consequent cardiac autonomic impairment and the well-acknowledged arrythmias and conduction abnormalities typical of CA [1]. Scintigraphy can be performed using Iodine-123-labeled metaiodobenzylguanidine (123I-MIBG), a chemically modified norepinephrine analogue, to detect an amyloid infiltration of the sympathetic nervous system. For this technique, semi-quantitative measurements of an LV tracer uptake can be applied, such as the heart-to-mediastinum ratio (HMR) or the washout rate (WR). SPECT can provide further information about the regional distribution of myocardial uptake [137]. In the AL subtype, HMR is reported to be lower than in healthy subjects but not as impaired as in ATTR-CA [137]. A correlation between these parameters and the occurrence of ventricular arrhythmias in patients affected by HF was previously reported [138] with an important prognostic implication. Cardiac sympathetic denervation assessed by MIBG imaging was demonstrated to be related to survival in ATTRv-CA [139]. Moreover, Piekarski et al. found that the decrease in MIBG uptake can be detected earlier than the evidence of higher DPD uptake in ATTRv-CA patients, supporting a diagnostic role for this tool in earlier identification of the disease [140]. PET tracers were also studied in this setting. There is evidence that Carbon-11 labeled meta-hydroxyephedrine (11C-mHED) is more accurate than 123I-MIBG in assessing regional sympathetic denervation in patients with LV dysfunction [141], but its role in the context of CA is unknown. However, the evaluation of sympathetic denervation in CA is a field of growing interest and new tracers for both SPECT and PET imaging are under development, but further studies and standardization of techniques are needed.

## 6. Cardiac Computed Tomography

Cardiac computed tomography (CCT) is a recently implemented cardio-radiological technique that is increasingly able to support clinical-therapeutic decisions even in extra-coronary contexts [120,142,143]. There is growing evidence about the frequent coexistence of ATTR-CA and AS, particularly concerning the low-flow low-gradient phenotype [144,145,146]. Treibel et al. reported a prevalence of ATTRwt-CA of 6% among AS patients aged > 65 years undergoing valve replacement surgery, with a demonstrated impact on patient survival, in agreement with subsequent research [144,147]. It is not fully elucidated whether this occurs because they are both diseases affecting aged subjects or whether AS is a consequence of the deposition of amyloid substance in the aortic valve. Most recent findings support the isolated amyloid infiltration of the aortic valve as a different process from CA in AS patients, with a possible contribution to worsening AS progression [148]. Castano et al. reported that 16% of patients with severe AS undergoing transcatheter aortic valve replacement (TAVR) were also affected by ATTR-CA [145]. The authors proposed a cutoff value of average tissue Doppler mitral annular S′ < 6 cm/s as a marker to perform a 99mTc-PYP scan and then continue testing for ATTR-CA. However, since computed tomography (CT) is always performed before TAVR procedures, its role in CA screening has been suggested in this population [145,148]. ECV values measured by pre-procedural CT with a 3 min acquisition protocol were demonstrated to be significantly higher in patients affected by ATTR-CA (then confirmed by scintigraphy), impairing patient prognosis and potential benefits of TAVR [149]. Since the kinetic properties of iodine contrast are similar to Gadolinium, CT can provide information such as ECV, late iodine enhancement (LIE), and myocardial perfusion, supporting its use even in patients who cannot undergo CMR. The disadvantage is that LIE has lower resolution than LGE, although novel CT techniques such as low-kilovolt scanning or dual-energy imaging can overcome this problem [150]. A delayed myocardial attenuation and relative attenuation index were demonstrated to be higher in CA patients than in controls [151]. In a study conducted by Chevance et al., CT-based ECV and LIE were significantly higher in CA patients than in controls and non-CA patients with LV hypertrophy [152]. The authors also found a decrease in mean myocardial blood volume and myocardial blood flow in the CA population, with a significant correlation with mortality. Similar to CMR-based ECV, Gama et al. [121] found higher ECV values in ATTR- than in AL-CA, with a good correlation with the grading of the disease and adverse myocardial remodeling parameters for both CA subtypes and with survival only for ATTR-CA. Also in this study, the ECV assessed by CT and by CMR were comparable, even using single-source 64-slice cardiac CT machines. In conclusion, CT is faster, cheaper, and more accessible than CMR and seems to have a good accuracy in CA diagnosis and prognosis, particularly through the measure of ECV, which is well correlated with CMR-based ECV. Further studies are needed to validate and standardize the use of this technique in the setting of CA and AS, with the definition of precise indications, parameters, and cutoff values.

## 7. Conclusions

A growing interest in CA, coupled with technological advances, has increased recognition of the pathology, which, however, remains underdiagnosed. Since efficient disease-modifying treatments have been introduced in recent years, an early diagnosis has the potential to definitively improve patient prognosis. Non-invasive diagnostic techniques are crucial for suspecting CA, and they can even lead to a definitive diagnosis for the ATTR subtype. Due to several studies performed in this field, these tools, ranging from ECG to multimodality imaging, are acquiring a growing accuracy not only in diagnosis but also in the prognosis and quantification of disease burden and response to therapy, as they are essential in guiding clinical decisions. Understanding new evidence regarding each of these methods is a key point in raising awareness about this often-underappreciated disease and in better managing CA patients, greatly improving quality of life, and overall survival.

## Figures and Tables

**Table 1:** Cardiac amyloidosis features at non-invasive diagnostic techniques.

ECG	Echo	CMR	Scintigraphy	PET Tracers	CT-Scan
Low QRS voltages	Left ventricle: symmetric hypertrophy, LVEF often preserved, MCF, SV, GLS, MW, SSR	LGE: subendocardial, transmural or patchy distribution, mainly in LV and RV	Perugini score	18F-florbetapir, 18F-florbetaben, 18F-flutemetamol	- Increased ECV
QTc(B), QRS-T-angle, III-QRS, aVF-QRS, and V3-R	Left atrium: LA strain depletion (reservoir–conduit-contraction), enlargement (LAVi)	Early enhancement (failure to null the myocardium on the inversion scout sequence): pathognomonic	Semiquantitative: - heart to contralateral lung ratio (1 or 3 h) - heart-to-thigh ratio - lung-to-thigh ratio - heart-to-whole-body ratio - heart-to-pelvis ratio	11-C-PIB, 123I-SAP, 124I-evuzamitide, 68Ga-FAPi	- Increased late iodine enhancement
- QRS I < 0.55 mV - QRS aVR < 0.5 mV - average QRS I + aVR < 0.575 mV - Average QRS I + aVR/average QRS V1–4 < 0.375 - Average QRS I + aVR/longest time to the onset of intrinsicoid deflection in leads I, aVL, V1–6 < 0.0115	Right heart: RV size, basal diameter, wall thickness, dilated inferior vena cava and right atrium, TAPSE, TDI S’ velocity peak, RV STE (reduced longitudinal free wall strain, RV apex-to-base strain gradient), TR	- Elevated native T1 values - Elevated ECV - Increased T2 times	Novel 3D-based quantitative indices: amyloid deposition volume, total amyloid uptake	Cardiac innervation: 123I-MIBG (heart-to-mediastinum ratio, washout rate)	- Increased relative attenuation index - Increased delayed myocardial attenuation
fragmented QRS		Emerging techniques: - Diffusion tensor CMR - Fingerprinting - CMR feature tracking (myocardial deformation)	Standardized uptake value max		
pseudo-infarction					
T wave inversion					
ST-segment depression					
conduction abnormalities					

LVEF: left ventricular ejection fraction; MCF: myocardial contraction fraction; SV: stroke volume; GLS: global longitudinal strain; MW: myocardial work; SSR: septal reflectivity ratio; LA: left atrium; LAVi: left atrium volume indexed; RV: right ventricle; TAPSE: tricuspid annular plane systolic excursion; TR: tricuspid regurgitation; LGE: late gadolinium enhancement; ECV: extracellular volume; 11-C-PIB: 11-C-Pittsburg compound B; 123I-SAP: 123I-labeled serum amyloid P component; 68Ga-FAPi: 68Gallium-labeled fibroblast activation protein inhibitor; and 123I-MIBG: Iodine-123-labeled metaiodobenzylguanidine.

**Table 2:** The most recent studies regarding different non-invasive diagnostic techniques for cardiac amyloidosis.

Study	Year	Technique	Type of Study	Sample Size	Description and Key Finding
Arana-Achaga et al. [8]	2023	ECG–Echo	Retrospective cross-sectional	227	T-Amylo prediction model for ATTR-CA (age, gender, carpal tunnel syndrome, IVSd thickness, and low QRS interval voltage).
Vereckei et al. [11]	2024	ECG	Retrospective cohort	79	Five novel ECG criteria tested for the diagnosis of CA.
Iijima et al. [12]	2023	ECG		50	New ECG scoring system for AL-CA.
Haimovich et al. [25]	2023	ECG	Retrospective	93,138	Artificial intelligence-based ECG model for detection and diagnostic classification of LVH.
Briasoulis et al. [39]	2023	Echo	Prospective	61	AL-CA patients with complete hematologic response to treatment showed a significant improvement in LV myocardial work indices at 1 year follow-up from treatment initiation, with improved survival.
Slostad et al. [45]	2024	Echo	Retrospective observational cross-sectional	167	The septal reflectivity ratio is a reproducible parameter with good accuracy in differentiating ATTR-CA from other phenocopies of CA and specifically AL-CA.
Bandera et al. [51]	2022	Echo	-	906	In ATTR-CA, significant infiltration of the atrial walls occurs, with progressive loss of atrial function and increased stiffness, which is an independent predictor of mortality.
Cai et al. [83]	2024	CMR	Meta-analysis (9 studies)	955	Higher ECV, T1 values, and lower T2 to skeletal muscle ratio were associated with heightened risk of mortality.
Dobner et al. [85]	2024	CMR	Prospective	51	Initiation of tafamidis preserved CMR-measured biventricular function and reduced LV mass at 1-year follow-up, whereas ECV and native T1-mapping did not change significantly.
Zheng et al. [93]	2024	CMR	Retrospective observational	139	LV torsion mechanics derived by CMR-FT can monitor the progression of cardiac systolic and diastolic dysfunction caused by amyloid deposition and are related to the LGE patterns.
Zhou et al. [98]	2024	CMR	Retrospective	120	LGE-based radiomic features are associated with all-cause mortality in CA patients, with better predictive performance than LGE semi-quantitative and quantitative parameters.
Ioannou et al. [77]	2023	CMR	Prospective cohort	221	Changes in native T1 in response to treatment are reflected in changes in T2 and ECV and in traditional biomarkers. Changes in native T1 are independently associated with mortality.
Guo et al. [104]	2024	Scintigraphy	Retrospective	54	99mTc-PYP scintigraphy has high diagnostic efficiency in ATTRv-CA and specifically 13Ala97Ser mutation. The semiquantitative parameter, H/CL ratio (cutoff value: 1.51), is more useful than visual score.
Gherghe et al. [108]	2023	SPECT/CT	Systematic Review (10 studies)	N/A	Quantitative SPECT/CT with 99mTc-labeled bone-avid radiotracers offers good prospects in the diagnosis and surveillance of ATTR-CA.
Matsuda et al. [114]	2023	Scintigraphy	Retrospective	32	Amyloid deposition volume and total amyloid uptake are two novel 3D-based quantitative parameters, which showed similar diagnostic and prognostic abilities to H/CL.
Tingen et al. [115]	2024	Scintigraphy	Retrospective cohort	32	Cardiac tracer uptake on bone scintigraphy reduces in ATTRv-CA patients treated with patisiran, but increases in the group treated with ATTR stabilizer. Bone scintigraphy is more sensitive of disease progression and treatment response as compared to the conventional follow-up parameters.
Yu et al. [116]	2024	SPECT/CT	Retrospective	13	The volumetric heart and lung ratio derived by SPECT/TC with 99m-Tc-pyrophosphate showed a significant reduction subsequent to eplontersen treatment in ATTRv-CA patients.
Ungericht et al. [99]	2024	SPECT/CT	Retrospective	28	In ATTR-CA, cardiac 99mTc-DPD uptake significantly correlated with histological amyloid load at endomyocardial biopsy.
Porcari et al. [117]	2024	SPECT	Prospective	1422	In ATTR-CA, biventricular tracer uptake was present in 100% of patients. Diffuse RV uptake (present in 66% of patients) is associated with poor outcomes and is an independent prognostic marker at diagnosis.
Genovesi et al. [118]	2021	PET	Prospective	60	Delayed [18F]-florbetaben cardiac uptake can discriminate AL-CA from either ATTR or other CA phenocopies.
Choi et al. [119]	2022	PET	Prospective	58	In AL-CA, 11C-PiB PET/CT adds incremental prognostic value to conventional serum biomarker and is a strong independent predictor of 1-y overall mortality.
Hayashi et al. [120]	2024	CT-CMR	Retrospective	31	Cardiac CT is comparable to CMR in quantifying myocardial ECV in CA, with significant correlation with clinical parameters.
Gama et al. [121]	2022	CT	Cohort	72	Cardiac amyloid burden measured by CT-derived ECV is associated with all-cause mortality in ATTR-CA patients.

ECG = electrocardiogram; CMR = cardiac magnetic resonance; SPECT= single-photon emission computed tomography; CT = computed tomography; PET = positron emission tomography; ATTR = transthyretin; CA = cardiac amyloidosis; IVSd = interventricular septum in diastole; LV = left ventricle; LVH = left ventricular hypertrophy; AL = light chains; ECV = extracellular volume; FT = feature tracking; LGE = late gadolinium enhancement; H/CL = heart-to-contralateral lung; RV = right ventricle; DPD = 3,3-diphosphono1,2-propanodicarboxylic acid; 11-C-PIB = 11-C-Pittsburg compound B.

## Data Availability

No new data were created or analyzed in this study.
